# Operatively treated ankle fractures in Switzerland, 2002–2012: epidemiology and associations between baseline characteristics and fracture types

**DOI:** 10.1186/s12891-021-04144-5

**Published:** 2021-03-11

**Authors:** Diogo Vieira Cardoso, Victor Dubois-Ferrière, Axel Gamulin, Christophe Baréa, Pablo Rodriguez, Didier Hannouche, Anne Lübbeke

**Affiliations:** Department of Orthopaedic Surgery, Geneva University Hospitals, Rue Gabrielle-Perret-Gentil 4, 1205 Geneva, Switzerland

**Keywords:** Danis-weber, Body mass index, Broos and Bisschop’s, Ankle fractures,malleolar fractures, Obesity

## Abstract

**Background:**

Ankle fractures are common, and their incidence has been increasing. Previous epidemiological studies have been conducted in the US, Scandinavia, and Scotland. Our objectives were to provide a current epidemiological overview of operatively treated ankle fractures and to evaluate the influence of age, sex, lifestyle factors, and comorbidities on fracture types.

**Methods:**

We performed a population-based epidemiological study of all ankle fractures treated operatively in a 10- year period from 2002 to 2012.

**Results:**

Two thousand forty-five ankle fractures were operated upon. Men and women differed significantly in age (median 41 vs. 57 years old), obesity (16% vs. 23%), diabetes (5% vs. 10%), smoking (45% vs. 24%), and accident type (daily activities 48% vs. 79%, transportation 24% vs. 9%, sports 21% vs. 8%) respectively. Overall, there were 2% Weber A, 77% Weber B, and 21% Weber C fractures; 54% were uni-, 25% bi-, and 21% trimalleolar; 7.5% of all fractures were open. Weber C fractures were much more frequent among men and with higher BMI (lowest vs. highest category: 14% vs. 32%), but slightly less frequent with older age and among current smokers. Trimalleolar fractures were twice as frequent in women and increased with age.

**Conclusion:**

Men and women differed substantially in age, lifestyle factors, comorbidities, accident type, and type of ankle fracture. Male sex and higher BMI were more frequently associated with Weber C fractures, whereas female sex and older age were associated with trimalleolar fracture. The risk for severe fracture increased linearly with the degree of obesity.

## Introduction

Ankle fractures in adults are common injuries, accounting for 10% of all fractures. Their incidence has been increasing since the 1950s; a recent study reported an incidence as high as 168.7/100,000 person-years [1, 2]. Population aging, increasing obesity prevalence, and more widespread participation in sports activities are thought to be the major causes [3]. Mean age at time of fracture was reported to be 41 years old. Ankle fractures are slightly more frequent in men than in women (53% vs 47%) [1]. Ankle fractures show a bimodal distribution with peaks among younger men and older women [1, 2].

Several classifications for ankle fractures are available [4, 5]. The Danis–Weber classification, proposed in 1972, is based on the level of the fibular fracture. It is frequently used by orthopaedic surgeons because of its simplicity and excellent inter-observer agreement [6]. Type A fractures occur below the syndesmosis, with the syndesmosis and the deltoid ligaments remaining intact. Type B fractures occur at the level of the ankle joint, and the syndesmosis complex can also be injured. Type C fractures occur above the syndesmosis complex and can consequently result in complete disruption of syndesmosis ligaments and an unstable mortise. Instability and syndesmosis disruption are associated with poorer function and early osteoarthritis [7] making type C fractures potentially more severe than type A or B [3]. In clinical practice, using Broos and Bisschop’s classification [8] according to the number of fractured malleoli is often useful to guide treatment decisions. Their uni-, bi-, or trimalleolar system is easy, intuitive, and has good prognostic value [9].

Using the Danis–Weber classification, the literature reports the fracture distribution as 3% type A fractures, 70% type B, and 27% type C. Unimalleolar fractures represent 55 to 70% of all fractures, bimalleolar fractures represent 20 to 24%, and trimalleolar fractures represent 10 to 23% [1, 9].

Previous epidemiological studies of ankle fractures have been conducted in the US, Sweden, Denmark, and Scotland, showing similar results despite geographic and temporal differences [10–13]. Although these studies investigated epidemiological data about ankle fractures, none of them explored associations between baseline population characteristics and fracture types using the Danis–Weber classification and the number of malleoli fractured. Moreover, to the best of our knowledge, this is the first epidemiological study of ankle fractures in Switzerland which spans a decade.

This study aimed to analyze the epidemiology of all the adult ankle fractures presenting at Switzerland’s largest trauma center and requiring surgery, from 2002 to 2012. It also aimed to identify associations between fracture types and age, sex, lifestyle factors, and comorbidities at the time of surgery.

## Material and methods

### Patients

The local ethics committee approved this study. We performed an electronic-chart review of every consecutive ankle fracture in patients aged 16 years old or more and treated surgically at Geneva University Hospitals, Switzerland, from January 2002 to December 2012. This is the largest level-1 trauma center in Switzerland.

All ankle fractures treated using open reduction and internal fixation (ORIF) were identified using the discharge diagnoses (International Classification of Disease codes S82.3 to S82.8) in the hospital’s information system. Data on patient’s baseline characteristics, including age, sex, comorbidities (American Society of Anesthesiologists or ASA score, and diabetes), and lifestyle factors (Body Mass Index or BMI, and smoking status) were obtained from anesthesia records. BMI was calculated by dividing patients’ weight (kg) by their height (m^2^). According to the World Health Organization, a BMI below 18.5 kg/m^2^ is considered underweight, 18.5 to 24.99 kg/m^2^ is normal, 25 to 29.99 kg/m^2^ is overweight, 30 to 34.99 kg/m^2^ is obese class I, 35 to 39.99 kg/m^2^ is obese class II, and above 40 kg/m^2^ is obese class III [14]. Mechanisms of accident were obtained from the emergency reports and categorized into simple fall, transportation accident (car, motorcycle, or bicycle), sports accident, and other types. Fracture patterns were obtained from surgical reports and grouped using the Danis–Weber classification. Fractures were further divided into unimalleolar, bimalleolar, and trimalleolar fractures, based on preoperative radiographs. The first author (DVC) analyzed all the radiographs, evaluating fracture patterns, and the presence of articular dislocation. To assess the interobserver reliability of the Danis–Weber and Broos and Bisschop’s classification, a co-author (PR) performed an independent analysis of 105 randomly selected cases.

### Statistics

Mean values and standard deviations were calculated for continuous variables, and frequencies and percentages were calculated for categorical data. To identify associations between baseline characteristics and fracture type, we performed separate uni- and multivariable logistic regression analyses for both fractures classified according to Danis–Weber and those classified by the number of fractured malleoli. The baseline characteristics considered were age (continuous), sex (m/f), BMI (continuous), ASA score (1–2 vs. 3–4), smoking status (current, former, never, unknown), and diabetes (yes/no).

The two-way random single measure intraclass correlation coefficient (ICC) was calculated to quantify interobserver reliability for the Danis–Weber and Broos and Bisschop’s classification. This is a special case ICC with a weighted kappa and has been considered equivalent [15]. Results were interpreted following Landis and Koch’s guidelines [16]. The statistical level of significance was set to *P* less than 0.05. Statistical analyses were performed using IBM SPSS Advanced Statistics, version 25.0.

## Results

The trauma center surgically treated 2045 ankle fractures. Mean patient age was 49 ± 18.4 years old. Men had 1032 (50.5%) fractures and women had 1013 (49.5%) (Table 1). Men were on average younger than women, 42 ± 16.8 vs. 55 ± 17.7 years old (*p < 0.001).* Men and women also significantly differed in BMI, with means of 25.8 ± 4.1 and 26.6 ± 5.6 kg/m^2^, respectively. Fifteen percent of men were obese (BMI ≥30 kg/m^2^) compared to 22% of women. Men and women also differed significantly (*p < 0.001*) in diabetes prevalence, 5 and 10%, and as current smokers, with 45 and 24%, respectively.

**Table 1:** Demographic and baseline characteristics (*N* = 2045)

	All	Women	Men
**Patients**	2045	1013	1032
**Age (years), mean (SD)**	49 (± 18.4)	55 (± 17.7)	43 (± 16.8)
**Lifestyle factors**			
**BMI (kg/m**^**2**^**), mean (SD)**	26.2 (± 4.9)	26.6 (± 5.6)	25.8 (± 4.1)
BMI by category (%)			
Underweight (≤ 18.5)	42 (2)	27 (3)	15 (2)
Normal weight (18.5–24.9)	832 (41)	391 (39)	441 (43)
Overweight (25–29.9)	682 (33)	318 (31)	364 (35)
Obese class I (30–34.9)	278 (14)	148 (15)	130 (13)
Obese class II (35–39.9)	69 (3)	48 (5)	21 (2)
Obese class III (≥ 40)	28 (1)	22 (2)	6 (1)
Missing	114 (6)	59 (6)	55 (5)
**Smoking status (%)**			
Current smoker	708 (35)	246 (24)	462 (45)
Former smoker	161 (8)	62 (6)	99 (10)
Never smoked	896 (44)	528 (52)	368 (36)
Not known if ever smoked	280(14)	177 (18)	103 (10)
**Comorbidities**			
**Diabetes (%)**	152 (7)	98 (10)	54 (5)
Missing	33 (2)	12 (1)	21 (2)
**ASA score (%)**			
I	692 (34)	267 (26)	425 (41)
II	1123 (55)	600 (60)	523 (51)
III	199 (10)	131 (13)	68 (7)
IV	12 (1)	8 (1)	4
Missing	19 (1)	7 (1)	12 (1)
**Fracture characteristics**			
**Right side (%)**	1103 (54)	544 (54)	559 (54)
**Open fracture (%)**	154 (8)	76 (8)	78 (8)
**Dislocated fracture (%)**	492 (24)	293 (29)	199 (19)
**Danis–Weber classification (%)**	1942 (95)	976 (96)	966 (94)
A	47 (2)	17 (2)	30 (3)
B	1491 (73)	796 (79)	695 (67)
C	404 (20)	163 (16)	241 (23)
Non-classifiable	103 (5)	37 (4)	66 (6)
**Number of fractured malleoli (%)**	2045 (100)	1013 (50)	1032 (50)
Unimalleolar	1099 (54)	431 (43)	668 (65)
Bimalleolar	519 (25)	283 (28)	236 (23)
Trimalleolar	427 (21)	299 (30)	128 (12)
**Type of accident (%)**			
**Simple fall**	1301 (64)	804 (79)	497 (48)
**Transportation**	333 (16)	91 (9)	242 (23)
**Sports**	293 (14)	82 (8)	211 (20)
**Other**	118 (6)	36 (4)	82 (8)

A simple fall was the most frequent accident type (domestic or daily activities, 64%), followed by transportation accidents (16%), sports accidents (14%), and other mechanisms (6%). Men and women differed significantly in type of accident: transportation and sports accidents were more frequent among men than women (transportation 23% vs. 9%, sports 20% vs. 8%, respectively), whereas simple falls were far more often the fracture’s cause among women (79%, compared to 48% among men).

Using a break-down by fracture type, Weber A fractures occurred in 47 (2%) patients, Weber B fractures in 1491 (73%), and Weber C fractures in 404 (20%). Unimalleolar fractures were the most frequent fracture type, with 1099 (54%), followed by bimalleolar with 519 (25%), and trimalleolar with 427 (21%). Of the total 2045 fractures, 154 (8%) were open fractures. Baseline characteristics in relation to the Danis–Weber classifications and to the number of fractured malleoli are presented in Tables 2 and 3, respectively. Interobserver reliability of two independent reviewers in classifying a random sample of 105 cases according to Danis–Weber resulted in substantial agreement, with an ICC of 0.809 (95% CI 0.730–0.866), and an ICC of 0.732 (95% CI 0.629–0.810) according to Broos and Bisschop.

**Table 2:** Baseline population characteristics in relation to the Weber classification (*n* = 1942)

	Weber A	Weber B	Weber C
**Age (years), mean (SD)**	37 (± 16.9)	50 (± 18.2)	45 (± 18.2)
**Men (%)**	30 (64)	695 (47)	241 (60)
**Lifestyle factors**			
**BMI (kg/m**^**2**^**),** (*missing = 106*), (%)			
BMI in categories (%)			
Underweight (≤ 18.5)	2 (5)	29 (2)	5 (1)
Normal weight (18.5–24.9)	24 (55)	603 (43)	149 (39)
Overweight (25–29.9)	14 (32)	511 (36)	133 (35)
Obese class I (30–34.9)	3 (7)	201 (14)	68 (18)
Obese class II (35–39.9)	1 (2)	45 (3)	20 (5)
Obese class III (≥ 40)	0	19 (1)	9 (2)
**Smoking status (%)**			
Current smoker	20 (43)	531 (36)	126 (19)
Former smoker	0	119 (8)	32 (8)
Never smoked	21 (45)	646 (43)	182 (45)
Not known if ever smoked	6 (13)	195 (13)	64 (14)
**Comorbidities**			
**Diabetes (**missing *n = 31*), (%)	2 (4)	120 (8)	29 (7)

**Table 3:** Baseline population characteristics in relation to the number of fractured malleoli (*n* = 2045)

	Unimalleolar	Bimalleolar	Trimalleolar
**Age (years), mean (SD)**	43 (± 16.7)	53 (± 19.2)	57 (± 17.7)
**Men (%)**	668 (65)	236 (23)	127 (12)
**Lifestyle factors**			
**BMI (kg/m**^**2**^**),** (*missing = 114*), (%)			
Underweight (≤ 18.5)	22 (2)	10 (2)	10 (3)
Normal weight (18.5–24.99)	462 (44)	205 (42)	165 (40)
Overweight (25–29.99)	360 (35)	172 (35)	150 (37)
Obese class I (30–34.99)	147 (14)	75 (15)	56 (14)
Obese class II (35–39.99)	35 (3)	17 (4)	17 (4)
Obese class III (≥ 40)	14 (1)	8 (2)	6 (2)
**Smoking status (%)**			
Current smoker	404 (37)	176 (34)	128 (30)
Former smoker	91 (8)	42 (8)	28 (7)
Never smoked	477 (43)	227 (44)	192 (45)
Not known if ever smoked	127 (12)	74 (14)	79 (19)
**Comorbidities**			
**Diabetes (%),** *(missing n = 33),* (%)	60 (6)	46 (9)	46 (11)

**Fig. 1:**
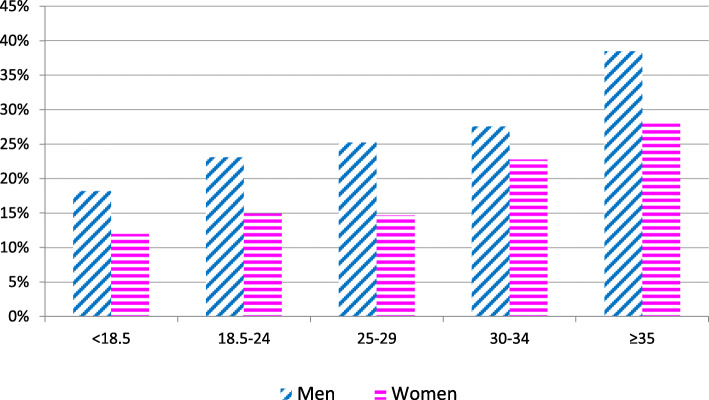
Proportion of Weber C fractures stratified by sex and BMI category

**Fig. 2:**
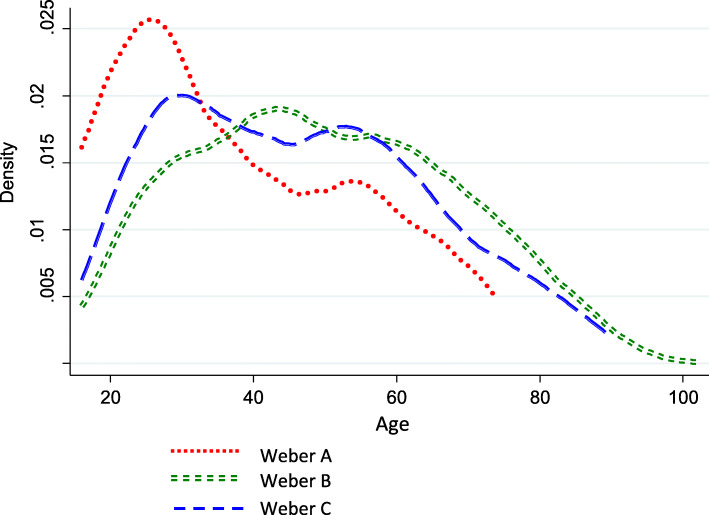
Age distribution at surgery according to the Danis–Weber classification

In both uni- and multivariable logistic regression analyses, Weber C fractures were more frequent among men (adjusted OR 1.59, 95% CI 1.23 to 2.05), in younger age groups (adjusted OR 0.990, 95% CI 0.982 to 0.997), and with higher BMI (adjusted OR 1.05, 95% CI 1.03 to 1.08) than were Weber A/B fractures (Table 4). The proportions of Weber C fractures by sex and BMI category are shown in Fig. 1. The increase in BMI-related risk was linear and observable in both men and women. The risk of a Weber C fracture was 14% in the lowest BMI category and 32% in the highest (taking men and women together). The age distribution at surgery in relation to the Danis–Weber classification is illustrated in Fig. 2. Weber C fractures were only less frequent among current smokers in the multivariable analysis (adjusted OR 0.75, 95% CI 0.57 to 0.99) (Table 4).

**Table 4:** Unadjusted and adjusted odds ratios (OR) for type Weber C vs. Weber A/B-type ankle fractures

	Unadjusted OR (95% CI)	Adjusted OR (95% CI)
**Age (cont.)**	0.99 (0.984–0.996)	0.99 (0.982–0.997)
**Sex (woman = 1)**	0.60 (0.483–0.753)	0.63 (0.487–0.811)
**BMI (cont.)**	1.04 (1.018–1.063)	1.05 (1.027–1.077)
**Smoking status**		
Never smoked	Reference category	Reference category
Current smoker	0.83 (0.650–1.080)	0.75 (0.574–0.991)
Former smoker	0.98 (0.645–1.6505)	0.88 (0.553–1.391)
Not known if ever smoked	1.16 (0.843–1.616)	1.38 (0.960–1.975)
**Diabetes (yes = 1)**	0.915 (0.600–1.394)	1.03 (0.643–1.665)
**ASA (ASA 3–4 = 1)**	0.73 (0.500–1.080)	0.80 (0.500–1.280)

**Fig. 3:**
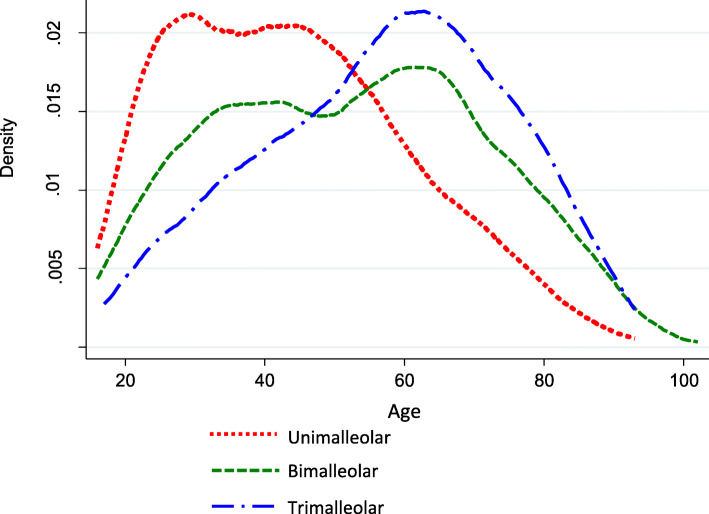
Age distribution at surgery according to the number of fractured malleoli

In both uni- and multivariable logistic regression analyses, trimalleolar fractures were more than twice as frequent among women (adjusted OR 2.3, 95% CI 1.8to 3.0) than were uni/bimalleolar fractures, and they increased with age (adjusted OR 1.03, 95% CI 1.02 to 1.03). Unadjusted and adjusted odds ratio and their 95% coefficient intervals for trimalleolar ankle fractures compared to uni/bimalleolar are shown in Table 5. Patients with ASA scores of 3–4 or diabetes had a higher risk of sustaining a trimalleolar fracture according to univariable analysis, but not after adjustment for the other factors. The age distribution at surgery in relation to the number of fractured malleoli is illustrated in Fig. 3.

**Table 5:** Unadjusted and adjusted odds ratios (OR) for trimalleolar vs. uni−/bimalleolar ankle fractures

	Unadjusted OR (95% CI)	Adjusted OR (95% CI)
**Age (cont.)**	1.03 (1.024–1.056)	1.03 (1.019–1.034)
**Sex (woman = 1)**	2.96 (2.352–3.719)	2.30 (1.782–2.979)
**BMI (cont.)**	1.01 (0.985–1.030)	0.99 (0.965–1.013)
**Smoking status**		
Never a smoker	Reference category	Reference category
Current smoker	0.81 (0.631–1.038)	1.13 (0.855–1.489)
Former smoker	0.77 (0.496–1.196)	0.81 (0.506–1.304)
Not known if ever smoked	1.44 (1.062–1.956)	1.36 (0.963–1.911)
**Diabetes (yes = 1)**	1.17 (1.198–2.482)	1.19 (0.780–1.839)
**ASA (ASA 3–4 = 1)**	1.54 (1.114–2.120)	0.74 (0.496–1.112)

## Discussion

The present study aimed to analyze the epidemiology of all adult ankle fractures requiring surgery in Geneva, Switzerland, from 2002 to 2012, and to describe associations between baseline patient characteristics and fracture types.

Our findings revealed significant differences between men’s and women’s ankle fractures. We found that male sex was more frequently associated with sustaining a Weber C fracture. Weber C fractures were also more frequently associated with higher BMI and less frequently with older age and current smoking status. Our results support the findings of Stavem et al. [9] who also reported a greater risk of Weber C fractures among men and with higher BMI. However, they found no associations with age or smoking status. King et al. [3] reported an association between younger age and more proximal fibular fractures, finding more Weber C fractures among patients below 25 years old than among older ones. They also reported Weber C fractures to be more frequent among men and patients with a BMI ≥30 kg/m^2^. These findings suggest a possible relationship between Weber C fractures and more physical and high-risk activities, which are more often associated with the young and male population.

Higher BMIs were also more frequently associated with more proximal fibular fractures. The relationship between obesity and more severe ankle fracture may be explained by the greater, weight-associated energy transmitted through the ankle joint during a fall. The association between obesity and ankle fractures has been reported before [17]. However, it is significant to note that, in our study, the relationship observed between higher BMIs and the increased risk of more severe fractures was linear and present in both men and women. This has not been reported before.

In contrast to men, who sustained more severe fractures at younger ages, we found that women and elderly patients were associated with an increased risk of sustaining a trimalleolar fracture rather than a uni- or bimalleolar fracture. Stavem et al. [9] reported similar results, finding that older age and female sex were associated with more fractured malleoli. This might be related to bone fragility and osteoporosis, as it is well known that aging and female sex are independent risk factors for this disease [18]. It has been suggested that ankle fractures among elderly women could be considered fragility fractures [1]. A Swedish study of adult ankle fracture epidemiology found that overall lifetime ankle fracture risk increased by 0.2% annually, whereas among women over 60 years old, the incidence increased by 0.9% annually [11]. This suggests that ankle fractures among elderly osteoporotic women are likely becoming more frequent as life expectancy rises. These fractures are challenging for orthopaedic surgeons as elderly populations are more fragile, with poorer pre-operative statuses and higher perioperative complication rates [11]. Furthermore, it is well known that the number of malleoli involved is directly related to higher mortality rates [19]. Some researchers have questioned whether ankle fractures can predict other osteoporotic fractures in women. The study by Pritchard et al. [20] showed that the occurrence of an ankle fracture did not predict any other major osteoporotic fractures in women. Therefore, the presence of an ankle fracture alone does not indicate a need for further osteoporosis assessment in women above 50 years old.

Although men and women differed significantly regarding the presence of diabetes mellitus (5% vs. 10%, respectively) at the time of surgery, our study found no associations between diabetes and ankle fractures. King et al. [3] and Stavem et al. [9] reported similar results. This contrasts with other studies suggesting that diabetic adults face an increased risk of sustaining ankle fractures, or indeed any type of fracture, even after adjusting for BMI [21, 22].

The most common fracture type in all age groups was a Weber B fracture, representing 73% of all fractures. Unimalleolar fractures represented the majority of fractures (54%), more than bi- and trimalleolar fractures together. Interestingly, 2% of the patients presented with a Weber A type fracture requiring surgical treatment. Although this type of fracture rarely requires surgery, ORIF of Weber A fracture can be performed if the fracture presents major displacement, in association with ankle dislocation and/or open fracture. Our results are in accord with other epidemiological studies of ankle fractures, indicating a similar distribution despite geographic and temporal differences [1, 3, 9].

The present study has several limitations. Firstly, the cohort was identified retrospectively via the hospital’s different data storage systems, using the discharge diagnosis, and some degree of diagnosis misclassification cannot be excluded. Secondly, both classification systems used in our study are purely descriptive and do not consider the mechanism of injury as proposed by other classification systems as Lauge-Hansen [4]. Also, not every ankle fracture reviewed could be grouped according to the Danis–Weber classification since it only considers lateral malleolus fractures. However, we decided to use the Danis-Weber and the Broos and Bisschop’s classification because, for many years, it was the primary classification system used for ankle fractures in our institution. Moreover, because of its simplicity, it is still often used among orthopedics surgeons around the world. Finally, our study only includes surgically treated ankle fractures, which might be a source of selection bias error towards more complex fractures. However, our results are in accordance with previous published results involving surgically and non-surgically treated ankle fractures [1, 3].

## Conclusion

This study found significant differences between ankle fractures sustained by men and women. Male sex and higher BMI were more frequently associated with Weber C fractures, whereas female sex and older age were associated with a higher risk of sustaining a trimalleolar fracture. Moreover, the risk of sustaining a more severe fracture increased linearly with the degree of obesity in both men and women. These findings, from a large recent cohort in Switzerland, confirm and complete previous publications from other countries.

## Data Availability

The datasets used and analysed during the current study are available from the corresponding author on reasonable request.
